# A Phase 2b Clinical Trial Assessing Lomecel-B Infusion in Individuals with Aging Frailty: Study Design and Rationale

**DOI:** 10.1093/geroni/igab046.2522

**Published:** 2021-12-17

**Authors:** Kevin Ramdas, Ben Hitchinson, Lisa McClain-Moss, Keyvan Yousefi, Liliana Diaz, Jorge Ruiz, Joshua Hare, Anthony Oliva

**Affiliations:** 1 Longeveron, Inc. , Miami, Florida, United States; 2 Longeveron, Inc, Miami, Florida, United States; 3 Longeveron Inc., Miami, Florida, United States; 4 VAMC, Miami, Florida, United States

## Abstract

Frailty is a common and important geriatric syndrome characterized by age-associated declines in physiology and function across multiple organ systems, which lead to increased vulnerability to adverse health outcomes. A biological mechanism that underlies the decline in physical function associated with aging frailty is chronic inflammation. The MSCs in Lomecel-B have immuno-modulatory capacity and control inflammation and the cytokine production of lymphocytes. An individual’s endogenous stem cell production decreases with age, this decrease likely contributes to reduced ability to regenerate and repair organs and tissues. Aging Frailty represents an exciting potential indication for cellular based therapies like Lomecel-B. This study is intended to evaluate the effects of Lomecel-B infusion compared to placebo on mobility and exercise tolerance, patient-reported physical function assessments and biomarkers for inflammation in individuals with Aging Frailty. This is a randomized, double-blind placebo-controlled, parallel multi-arm multicenter study enrolling adults aged 70-85 years identified as mildly or moderately frail per the CSHA Clinical Frailty Scale (CFS), with reduced six minute walk test (6MWT) and elevated Tumor Necrosis Factor-α (TNF-α), at screening. 150 subjects (30 per group) were randomized to receive a single peripheral intravenous infusion of 25, 50, 100, or 200 million doses, or placebo. Safety and efficacy assessments were conducted at 30, 90, 180, and 270 days after infusion. A follow up telephone call to subjects was placed at 365 days. We describe the design and rationale in detail of this 2b study assessing the effects of Lomecel-B on older adults with Aging Frailty.

